# The Medical Council

**Published:** 1862-01

**Authors:** 


### MEDICAL CRITIC

AND

### PSYCHOLOGICAL

### JOURNAL.

JANUARY, 1862.

### Art. I.—THE MEDICAL COUNCIL.

The General Council of Medical Education and Registration has hitherto enjoyed an existence above and apart from the storms of criticism. There has been a very general feeling that the members should be allowed to define the boundaries of their official duties, and to give practical evidence of their capacity, or incapacity, for discharging them, before either praise, censure, or suggestion were permitted to add to the difficulties inseparable from a position so entirely novel, and concerning which so many vague expectations had been aroused. It will readily be admitted, we think, that the profession may now, with entire propriety, abandon this attitude of expectant attention; and, from the materials before it, may both pronounce a just judgment upon the past, and form a definite conception of the probable future.

In one of Lord Macaulay's graphic essays, he has left a description of the results, political, moral, and social, that were expected to follow the fall of Walpole from power, and the accession of his rival, Pulteney:—"It was Pulteney's business to abolish faro and masquerades, to stint the young Duke of Marlborough to a bottle of brandy a day, and to prevail on Lady Vane to be content with three lovers at a time." The Medical Act, and the operations of the Medical Council, have served many interested persons as the bases of anticipations not more or less unreasonable. Quackery would cease. The "counter-practice" of druggists would be abolished. The sale of medicines, except by the dispensing of authorized prescriptions, would be made a penal offence. Grandmothers would no longer recommend specifics against hooping-cough or measles; and Ladies Bountiful and the clergy would stand respectfully aloof from the awful mysteries of physic. Some hardy speculators even looked forward to a time when the profession would be "true to itself;" or, in other words, to the fabrication, from all doctors, of a corporate Esau, whose hand should be against every man, and who should place Boards of Guardians, clubs, and patients generally, under the yoke of a medical tyranny the like of which the world had never before witnessed.

"Whatever the people wanted," proceeds Lord Macaulay, "they certainly got nothing. Walpole retired in safety; and the multitude were defrauded of the expected show on Tower Hill. The Septennial Act was not repealed. The placemen were not turned out of the House of Commons. Wool, we believe, was still exported. 'Private life' afforded as much scandal as if the reign of Walpole and corruption had continued; and 'ardent youth' fought with watchmen and betted with blacklegs as much as ever."

It is painful to be obliged to continue the parallel, and to confess that our brethren do not appear to have realized any one of the objects of desire discoursed of at Branch Association meetings, or promised in weekly journals. Quackery is still rampant;—domestic medicine still flourishes. Druggists sell pennyworths of jalap, and, worse still, pennyworths of laudanum, without having the fear of the law before their eyes. They even continue mysterious confabulations with dyspeptic customers; confabulations in which "the wind" and "constitution" are the leading ideas, and which eventuate in the preparation and consumption of many pills. Mr. Pestle, L.A.C., complains bitterly of the registration fee as a sheer robbery; and says, with much semblance of truth, that, having paid it, he is no better off than he was before.

What, then, is the object of the Medical Act? and what are the functions of the Medical Council? The former is said to be, in the preamble, "that persons requiring medical aid should be enabled to distinguish qualified from unqualified practitioners;" and the latter are thus explained by the Council itself, in a letter addressed to the Secretary of State:—

1. "The Council is called on to exclude all irregular practitioners from the Register, and *it* has exercised the greatest possible care in discharging *that* part of its duty.

2. "The Council is called on to expunge from the Register the name of any unqualified person who may obtain admission by false pretences. This power has been already exercised.

3. "The Council has *the* power to expunge from the Register the name of any practitioner who may be found guilty, in England or Ireland, of a felony or misdemeanour; or, in Scotland, of a crime or offence. *One offence of this nature has occurred,* but the name of the convicted person having been expunged from the Register for another cause, the Council need not proceed in the matter.

4. "The Council has *the* power to expunge from the Register the name of any practitioner whose name may have been struck off from the list of the members of any of the bodies which grant medical qualifications.

"It is no part, however, of the functions of the Council, according to the Act, to institute prosecutions at large for offences against the Act."

Now, upon the sole showing of the above modest and curiously constructed sentences, it appears to us that the Council is only moderately useful, while we know that it is very dear. An intelligent clerk would perform with perfect accuracy the functions detailed in the numbered paragraphs; and indeed, for those referred to in Nos. 3, and 4, intelligence is scarcely needed. We presume, of course, that *all* convicted persons, and *all* persons deprived of their qualifications by the bodies which originally granted them, are intended to be struck off the register; and, although we know at least one medical convict whose name is still retained thereon, we believe this to depend upon inadvertence on the part of the Council, and not to be designed either as a precedent or as an invidious distinction. The fact of conviction, communicated, as it should be, by prison authorities, or the fact of removal from a list of licentiates, communicated by the secretary of the licensing body, ought in every case to be followed by corresponding alterations in the Register. The powers and duties described in paragraphs 1 and 2 might, perhaps, if entrusted to a single clerk, expose him to occasional imputations of partiality or error. An appeal to the nearest police magistrate or county court judge would, however, effectually dispose of such imputations at a very moderate cost. The Medical Council consists of practising physicians and surgeons of the highest eminence, whose time is almost public property, whose moderate fees for attendance amount in round numbers to a hundred and thirty guineas for each day of meeting, and whose deliberations and decisions, on the question whether John Smith has told a lie to get a place in the Register, exhibit the most absurd invocation of Hercules, the most ludicrous disproportion of means to an end, with which our experience of the world has hitherto made us acquainted.

About many small matters, however, it would seem that the Council has been more than once perplexed; and its perplexity has invariably issued in the adoption of the familiar maxim of

Captain Lutteridge, "Sometimes, it seems, things will so turn out that no man can possibly know how to act; and then, the only thing is, to do NOTHING, which can never be wrong." As examples of this philosophy we take the following extracts from the minutes; repeating our former observation, that the results might have been obtained by a more simple and less expensive machinery.

*August 5th*, 1859.—Amendment moved by Mr. Syme, seconded by Mr. Lawrence, and agreed to—

"The Council having heard the statements of Dr. Apjohn, Dr. Williams, and Dr. Stokes, on their respective sides of the question relative to the Surgical Licence of Trinity College, Dublin, and the opposition of the Royal College of Surgeons of Ireland, *deem it inexpedient to express any opinion on the subject in dispute.*"

*June 14th*, 1860.—Letters having been read, addressed by the Metropolitan and Provincial Inspectors of Anatomy to the President of the Medical Council, suggesting that, with a view to the better working of the Anatomy Act in England, the Council should recommend that the Medical Session in London should commence on the first of November instead of on the 1st of October in each year,

Resolved—"That answers should be returned, stating that *the subject does not lie within the province of the Medical Council.*"

"A letter having been read from Mr. William Whitehead Morris, complaining that he had been threatened with prosecution for assuming the title of surgeon, he being a Licentiate of the Apothecaries' Society of London, and not possessing a surgical diploma, it was directed that an answer should be returned, stating that the subject of his communication *is not a matter in which the Medical Council can interfere.*"

*June 22nd*, 1860.—Letters were read from Mr. William Gwynn, stating that the University of Dublin had refused to admit him to the degree of Doctor of Medicine, and to that of Master in Surgery, on the ground that he had adopted a particular theory of medicine, and requesting 'that the Medical Council, in accordance with the Act 21 and 22 Vict., cap. 90, sec. 23, will represent the same to Her Majesty's Most Honourable Privy Council.

Moved by Mr. Syme, seconded by Dr. Andrew Wood, and agreed to—

"*That the Council resolve that they see no reason for taking any step in relation to the letters of Mr. Gwynn.*"

A reference from the Royal College of Physicians of Edinburgh, in regard to the ordinances of the University (Scotland) Commissioners for conferring degrees in Medicine in the University of Edinburgh, having been read,

Moved by Dr. Alexander Wood, and seconded by Mr. Lawrence—

"That the General Council of Medical Education and Registration having taken into consideration the ordinances of the Scottish University Commissioners, for regulating the conferring of medical degrees in the University of Edinburgh, dated 6th August, 1859, which were laid before them by Mr. Syme on the 8th of August last, and also the further supplementary ordinances of the 19th March, 1860, which have been circulated among the members of the Council, find :—

1. "That having already declared that the higher degrees in medicine should be distinguished by corresponding academic rank, obtained by a full, and as nearly as possible equivalent, academic education, they are compelled reluctantly to record their opinion that these ordinances, if carried into effect, will tend to frustrate their efforts in this direction.

2. "That having also declared 'That all students should pass an examination in general education before they commence their professional studies,' the Council are of opinion that it is impossible for the generality of students to acquire, before the age of seventeen years (which they must do, if they are to take their medical degree at twenty-one years of age), such a general education as shall enable them to prosecute their medical studies with success, and afterwards to take the position which university graduates ought to take among the educated classes of the community.

3. "That, in the opinion of the General Council, the scheme proposed by the Commissioners, by which a degree in medicine and a degree in surgery are to be given after one course of study and one examination, tends inevitably to establish a fictitious, not a real distinction, between a physician's and a surgeon's diploma, and is opposed to the spirit of the Medical Act."

Amendment moved by Mr. Syme, and seconded by Dr. A. Thomson—

"That it is inexpedient for this Council to express any opinion at present with respect to the terms upon which degrees in medicine are conferred by the University of Edinburgh."

Amendment negatived.

Dr. Alexander Wood required that the names of the majority and minority should be entered on the minutes.

*Majority*—Dr. Burrows, Mr. Green, Dr. Acland, Dr. Bond, Dr. Embleton, Dr. Andrew Wood, Dr. Alexander Wood, Mr. Watt, Dr. A. Smith, Dr. Apjohn, Dr. Corrigan, and Mr. Teale.

*Minority*—Mr. Nussey, Dr. Storrar, Mr. Syme, Dr. A. Thomson, Dr. Leet, Sir Charles Hastings, and Dr. Christison.

The original motion was then put, and *also negatived.*

Dr. Alexander Wood required that the names of the majority and minority should be entered on the minutes.

*Majority*—Dr. Burrows, Mr. Nussey, Dr. Acland, Dr. Bond, Dr. Storrar, Mr. Syme, Dr. A. Thomson, Dr. Leet, Sir Charles Hastings, and Dr. Christison.

*Minority*—Mr. Green, Dr. Embleton. Dr. Andrew Wood, Dr. Alexander Wood, Mr. Watt, Dr. Smith, Dr. Apjohn, Dr. Corrigan, and Mr. Teale.

The subject then dropped; and the Council proceeded to other business.

On Friday, June 28th, 1861.—A motion for the admission of reporters for the press to the meetings of the General Council was negatived, the proposer and seconder only (Drs. Andrew and Alexander Wood) voting in its favour.

The reader, we apprehend, will hardly have followed us thus far without perceiving that the Council has deliberately rejected the only means by which the performance of the routine devolving upon it could be elevated into action at all worthy of the distinguished position of the individual members. The complaint of Mr. Morris, although not a matter in which the Council could interfere, afforded an opportunity for any councillor to express an opinion, which the profession would have received with respect, about the absurd intention then generally entertained, as the result of exasperating and perhaps unscrupulous local competition, to subject licentiates of the Hall to legal persecution at the hands of their doubly qualified brethren. A few sentences of plain and emphatic condemnation from almost any member of the council, and the general assent which those sentences would necessarily have commanded, would have rendered the intention one that no outwardly respectable practitioner could afterwards have ventured to avow. The application of Mr Gwynn, who is, we suppose, a homœopath, would have rendered it easy to make public the opinions of the Medical Council on the conduct of men who practise that quackery under the shield of examinations passed before, and qualifications conferred by, any of the bodies whose business it is to license the professors of legitimate medicine or surgery. But, for the fitting use of these opportunities, the one thing needful is publicity.

Proceeding to matters still more important, such as the debate about the differences between Trinity College and the Irish College of Surgeons, or about the regulations of the Edinburgh University, it is manifest that the grounds of judgment are fully as important as the decision arrived at. In the latter instance especially, when, after an elaborate motion and an amendment absolutely opposed to that motion had both been negatived, the Council suffered the matter to drop, and arrived by a circuitous route at Captain Lutteridge's conclusion, what were the motives of action in the case? and what were the grounds of difference? Were the divisions fair? or the results of a cross? Were some of the opposing councillors mere delegates, supporting the interested views of their constituencies? were others influenced in their votes by considerations of expediency, and a desire to temporize, or by definite opinions about the principles involved? Such are some of the questions that must suggest themselves to every reader of the barren minutes from which we quote—questions that will never be satisfactorily answered until the journals can place verbatim reports of the council meetings in the hands of every one who cares to read them. If such reports were regularly published, the Medical Council could not fail to exert, even by its incidental discussions of the various topics of professional interest that now and again float to the surface, a very great and beneficial influence both upon practitioners and upon the public. The sense of responsibility under which speeches would be made, and votes recorded, would invest the proceedings of the Council with dignity and importance—attributes which closed doors must for ever shut out. On the system that has hitherto been adopted, there are probably few practitioners who know that the Council has ever met at all, or, if meeting, what it has done; and a continuance of this system, by rendering the Council either a cumbrous machine for the registration of foregone conclusions, or a mere tool to be fought for by the secret cabals and intrigues of rival corporations, will speedily reduce it to a cipher, doomed to perish of its own insignificance, or to be swept away by the next wave of medical reform. In these critical and irreverent days, something more than great names and ungrammatical resolutions are needed, in order to obtain confidence, and with confidence stability, for a new and untried deliberative body; and the Council may be quite sure that, however sound their conclusions and however judicious their actions, those conclusions will be questioned, and those actions misjudged, unless the grounds on which they rest are, from time to time, placed fully and clearly before the profession in the pages of the medical journals.

We should do the Council injustice, however, if we gave them credit for no more work than that set forth in the modest programme we have quoted. They have further undertaken three Herculean labours—namely, to reform medical education, to compose a National Pharmacopœia, and to frame an amended Medical Act.

In the first of these matters, the reform of medical education, the Council have actually made very considerable progress; and have ensured for themselves the gratitude of every well-wisher to the profession. For many years past it has been painfully evident that the great body of general practitioners wanted, not so much the mere information or knowledge imparted by what is called a liberal education, as the habits of mind resulting from early and systematic training of the intellectual faculties. Well-meaning but mistaken men, in their anxiety to open a door for the cheap and easy attainment of medical qualifications, have very perceptibly lowered the social and intellectual status of the whole profession; and while a few eminent minds have everywhere enlarged the sphere of medical and surgical labours, there has been a slight, but steady and manifest, deterioration on the part of the labourers. It is impossible to read a medical journal, or attend a medical meeting, or, after consultation with a country practitioner, to question him about his professional neighbours, without obtaining evidence of the existence of a widely diffused feeling of personal jealousy and spitefulness, almost feminine in some of its characteristics, and only possible among persons whose emotions are accustomed to exercise sway over their judgments. To the action of the Council it is due that this condition of things is doomed. The licensing bodies chiefly resorted to by general practitioners have been accustomed either to confine their attention to professional subjects, as in the case of the English College of Surgeons; or to be content with a superficial classical examination at the same time as the professional one, as in the case of the London Apothecaries' Company or the University of St. Andrew's. For the last named bodies, it was a common practice for students, whose general education had been entirely neglected, to grind up a little Celsus during their last weeks of preparation, and to obtain their licence without having ever received the training which their carelessly tested knowledge was supposed to denote. For the future, by regulations of the Council now universally known, a fairly good preliminary education must in every case be received, and shown by examination, before the study of professional subjects can be commenced; and we hope that the standard of this preliminary education will be steadily raised as time proceeds. The Council have not the credit of discovery or originality in the matter; for the necessity of such a regulation has been often and often pointed out; and all we can know of their opinions is that, upon the whole, they recognise the fact of this necessity. How satisfactory would it have been if the councillors had stated the grounds of their opinions, and had reviewed and disposed of the possible objections to their scheme. How satisfactory to have heard, from the venerated President of the Royal Society, the conclusions about medical education to which his magnificent intellect has been guided by his vast experience. Here and there faint and feeble voices have been raised, complaining that the expense of the new system, and the refinement of mind to be expected from the new generation of practitioners, are calculated to cut off the present abundant supply of doctors willing to work cheaply among the poor. Surely there are those in the Council who would have said with authority, if their voices could have been heard without the walls, that no man of uncultivated mind can possibly grasp the problems daily presented by pathology and therapeutics, or can ever be safely trusted with the weapons of the medical armoury. Surely there would have been abundant testimony to the fact, daily illustrated by the great body of our parochial clergy, that men of the highest cultivation and refinement give to the poor an amount of friendliness, of sympathy, and of unrequited labour, such as not even the writers in the *Lancet* could venture to attribute to the licentiates of the Apothecaries' Company. By votes and resolutions the Council may influence the letter of educational regulations; but by open discussion it would influence, or even absolutely sway, the convictions of the public.

The remaining matters, the Pharmacopœia and the Medical Act, are exclusively of medical interest. The former, it was said, would be ready for issue in November, 1860, and the Committee engaged about it even tied themselves to time so far as to specify the "middle or latter end" of that month as the time of appearance. Another year has passed away, and there is no Pharmacopœia. We do not regret the delay; for the compilation of a Pharmacopœia is a matter of conventional necessity rather than of practical utility. It is doubtless well to have such a standard; but the completed volume must always be in arrear of the pharmacy of the day. We hope, therefore, that the compilers will be anxious to do their work well, rather than to complete it quickly.

The amendment of the Medical Act is a subject that has been before the Council both in 1860 and in 1861; and that has been referred to and reported upon by a special committee in each year. We are disposed to agree with these Committees in the view that more time, and more experience of the present Act than has yet been gained, are required as preliminaries to successful legislation. There is a very general demand for stringent penal clauses against unqualified practitioners—a demand in which we cannot join. We would elevate the status of the qualified practitioner in every possible way, both educational and social, and would then leave him to fight against the quack with the weapons of superior skill and knowledge. We would carry out the preamble of the present Act thoroughly, and enable those requiring medical aid to distinguish between qualified and unqualified practitioners; and we would even deprive the unqualified practitioner of any status in a court of law, whether for goods supplied or work done. But, as far as we can judge at present, we would not persecute the quack into importance, or condescend to accept, for the profession as it will be, a protection that is scarcely needed even for the profession as it is. On the whole subject of the relations of medicine to quackery, the opinions of the Council might be reported with great advantage. The vulgar commonly suppose that the doctor regards the quack as a rival, and wishes to crush him, out of jealousy or from motives of gain. The publication of a single debate would show that physicians and surgeons of sufficient eminence to be raised above all rivalry from quacks regard them, not with the jealousy of competitors, but simply with the feelings that honest men entertain towards thieves.

We think, however, that there can be no doubt about the advantages that would result from certain changes in clause 29, or from an authoritative and extended interpretation of the intentions of the Legislature in forming it. This clause, after permitting the Council to expunge from the Register the name of any person who may be convicted, in England or Ireland, of a felony or misdemeanour; or, in Scotland, of a crime or offence, proceeds, " If any registered Medical Practitioner shall, after due inquiry, be judged by the General Council to have been guilty of infamous conduct in any professional respect, the General Council may, if they see fit, direct the Registrar to erase the name of such medical practitioner from the Register." The fact of conviction is at least a well-defined basis of procedure; but the additional clause seems to provide for the punishment, by a secret and irresponsible tribunal, of an offence previously unknown to law. A Mr. John Kearney has already suffered under this clause; and we presume that no one, his friends and neighbours excepted, have any knowledge of the charge brought against him, or of the nature of the evidence by which he was condemned. It is plain that a punishment thus inflicted cannot serve as a warning to others who may be inclined to repeat his conduct.

Now, in order that this clause may work efficiently, we think not only that the meaning of the words "infamous conduct" should be clearly specified, but that the charges should be heard in open court. We think, also, that the Council should have a power of suspension from the Register, to meet cases for which the extreme penalty would be too severe.

What is meant by "infamous conduct in any *professional respect?*" Would the seduction of a patient come within the scope of the words, or the seduction, by a medical assistant, of his employer's wife, or daughter, or visitor, or servant? These questions alone are sufficient to show the difficulties by which the matter is beset, and the differences of opinion likely to arise about it. Would gross ignorance and malpractice be infamous in a professional respect? For our own part, we should answer in the affirmative, and should rejoice to see the guilty persons removed from the profession. The examination of a licensing body is but an imperfect test of fitness to practise; and the only real test is applied by the events of life. We would allow the latter to reverse conclusions based only upon the former, and, under whatever name denounced, we would have the ignorant or the careless deprived of the status afforded them by a legal qualification. Habitual drunkenness is infamous enough, and is, perhaps, the vice that most frequently brings its medical victims within the grasp of the law; but is it infamous *in a professional respect?* and could the Council disqualify a sot when, as yet, he had not disembowelled a parturient woman, or left a patient to die of undetected hernia? On these and on some kindred matters the Act, when amended, should speak clearly.

Among alterations of detail, however, that might justifiably precede a general measure, we would suggest that power should be given to the Council to appoint a writer of English at a reasonable stipend, whose duty it should be to translate motions, amendments, and reports, out of the dialects in which they are framed, into the vulgar tongue, before they are either put to the vote or published in the minutes. We have been much struck with one amendment, that was agreed to solely in order to render a previous motion intelligible; and much also with the following curious passage, which we publish, without comment, further than to explain that the words italicised are not intended to relate to the registration of the Registrar himself, but to that of some other individual. A Committee of Council, reporting upon the method of entering names in the Register, says—

"The Act clearly affords liberty for entrance on the Register in any one of the three following ways:—1st. Production to the Registrar of the Branch Council of the document conferring or evidencing the qualification. 2nd. *Transmission by post to such Registrar of information of his name and address, and evidence of the qualification or qualifications in respect whereof he seeks to be registered, and of the time or times at which the same was or were obtained.*"

Shade of Cobbett! If the councillors speak as they write, we cease to wonder that reporters should be excluded.

